# Migrants in Chile: Social crisis and the pandemic (or sailing over troubled water…)

**DOI:** 10.1177/1473325020973363

**Published:** 2021-03

**Authors:** Vanessa Jara-Labarthé, César A Cisneros Puebla

**Affiliations:** Escuela de Trabajo Social, Universidad de Tarapacá, Arica, Chile

**Keywords:** Crisis, migration, social policy, migrants in Chile, vulnerability, Covid-19

## Abstract

Chile has sailed in troubled waters in recent months. The effects of the social crisis at the end of 2019 were not yet fully evident, when the Covid-19 pandemic forced the government to take drastic measures to try to slow down the advance of the virus. The restrictions imposed on displacement, dynamic quarantines and the suspension of non-essential activities had a strong impact on the employment and economic conditions of the inhabitants of Chile, and more dramatically on the migrant population. This article aims to make visible the vulnerability and precariousness of the migrant population in Chile in this context of a pandemic, as well as the need to generate situated and inclusive social policies.

2020 is now a very meaningful number. It is a year that combines - as never seen before - apocalyptical visions, conspiracy theories, fake news, and horrendous scenes for the future after the coronavirus pandemic. Since the very beginning, news was so alarming that necropolitics (Mbembe, 2003) became a popular term amid any collective concern about the health policies created around the world. Theoretical ideas about the cyclical crisis of capitalism were also on everyone's lips again remembering the beginning of 1929 recession and the total deaths caused by Spanish flu since 1918.

Regardless the data from Europe, Asia and North America, countries such as Mexico, Brazil, Ecuador and Chile have been in critical periods due to the number of infections and deaths caused by the coronavirus. The world alarm focused on vulnerable people in any country due to disease conditions, age, and being outside the reach of health systems. Undoubtedly, all the miserable, homeless, hobos, poor inhabitants of the streets of big cities, the rural poor, landless peasants, neighbors far from medical care centers, unemployed people and migrants would be the first to die as a result of the pandemic.

Chile has a place in the world: it is the country in which the most liberal economic policies have been implemented, taking place the most spectacular free market experiment, together with the United Kingdom. Such an experiment began with the massacre that ended the socialist regime of President Salvador Allende, who was democratically elected in 1970. On September 11, 1973, with a criminal coup, Pinochet began one of the most terrorist dictatorships in human history. Military dictatorship that ended in 1990 to give way to a “concerted democracy” that is actually a civil dictatorship that has had Aylwin, Frei, Lagos and Bachelet as presidents. Nowadays this civil dictatorship it is headed by Sebastián Piñera. In the last 30 years (1990–2020) Chile has been named as “Chilean way of life” for the migrants, in reference to the “American way of life” for other migrants; or “oasis” in the midst of convulsed South America. However, as Gaete et al. (2019) among other voices have written: “… only 1% of the population has ended up concentrating 33% of national wealth, while 80% has suffered the indolence of a state that does not act as a guarantor of social security nor essential services …” (p. 1).

In recent years, Chile became a main destination for migrants seeking to improve their living conditions. According to the National Statistics Institute (INE, 2019: 4) “in the last 5 years, the number of foreigners in Chile has almost tripled, increasing the presence of two non-border countries: Venezuela and Haiti”. The increase of migrant population involves not only the increase of the population living in the country, but also challenges for the dynamics of the cities and living conditions of the people.

By December of 2018 there were a total of 1,251,225 foreigners living in Chile, with 51.6% men and 48.4% women (INE, 2019: 29). The most prevalent community was the Venezuelan with 288,233 people, a position that the Peruvian population occupied for decades. The second larger community was Perú with 223,923 people; then Haiti with 179,338; Colombia, 146,582 and Bolivia with 107,346 people (INE, 2019: 31). Following the same report, almost 60% of the estimated migrant population is concentrated between the age of 20 and 39 years old, which may indicate that they are part of the potentially economically active population.

According to the place of residence, by 2017, the Metropolitan region concentrated the highest percentage of migrants at the national level (69.1%), but looking closely the relation of amount of migrants and the total of population, it is in the north where the highest values are displayed; for example in the region of Tarapacá, where the migrant population amounts to 9.4% of the regional total (National Foundation for Overcoming Poverty, 2017: 15). In relation to gender distribution, the metropolitan region had similar percentage between men (49,1%) and women (50,9%), but “in the northern macrozone - which includes the regions of Arica and Parinacota, Tarapacá, Antofagasta and Atacama- there is a greater presence of women (56.7% of women versus 43.3% of men)” (National Foundation for Overcoming Poverty, 2017: 16).

In Chile, the pandemic of COVID-19 was officially declared by March 3, 2020 after the confirmation of the first case. Soon after the first case, the virus spread across the country, slowly at the very beginning. By March 18^th^, Chilean President Sebastian Piñera declared for second time the Constitutional State of Exception for 90 days, followed by several preventive measures to face the pandemic in areas such education, health, transport, borders, among others. Regardless the national socialist origin of the term “State of Exception”, in Chile is constitutionally enshrined as the President´s right to declare that individual liberties are restricted by public calamity. The first time he promulgated State of Exception was on October 19, 2019 because of the largest and most splendid manifestation of popular discontent in the recent history of Chile.

In relation to education, all educational and pre-school institutions were closed, to prevent any potential increase of infections. For the case of borders, at the beginning of the pandemic, the actions established that all people coming from countries classified as high risk by the World Health Organization, had to be in a quarantine of 14 days upon entering the country. However, by March 18, 2020 all Chilean land, sea and air borders were closed for the transit of foreign people.

One of the most important measure was the establishment of dynamic quarantines across the country which limited the freedom of movement: only essential businesses like grocery stores and pharmacies were allowed to stay open, and most people, except essential workers, were expected to stay home. However, these measures highlighted the precariousness, overcrowding and poverty of migrant populations along the country.

It is not a mystery that migrants often must face discrimination, poor living conditions, social exclusion, informal and precarious employment, among other issues that, in this current pandemic context, reinforces their condition of vulnerability. At this point, it is possible to state that “migrants’ specific patterns of vulnerability often lie at the intersection of class, race and status: migrants are overrepresented in low-income and discriminated minorities” (Guadagno, 2020: 5).

Work, housing, health and even life support have entered into crisis for the migrant population: after the establishment of quarantines in the country, they have been forced to risk - or even to lose - the stability that many had achieved during their life in Chile. The first effect was the increase of the precariousness of their working conditions or the unemployment, followed by their potential inability to pay housing rentals or even to buy food.

According to data provided by the National Foundation for Overcoming Poverty (2017), migrant population was mainly employed in activities associated with “commerce (20.7%), hotels and restaurants (12.6%), domestic service (12.3%) and construction (11, 4%). The main occupational category corresponds to a private sector employee or worker (69.6%), followed by that of self-employed worker (14.8%) and door-to-door domestic service (6.5%)” (National Foundation for Overcoming Poverty, 2017: 17). However, it is a fact that in many cases, working relations are informal, without an employment contract or social benefits, without stability and with great precariousness, which leaves the migrant population in a vulnerable condition, without real or effective protection mechanisms that allow them to face a crisis like the one we are experiencing due to the pandemic.

In relation to overcrowding, once migrants arrive in Chile, “higher degrees of residential segregation, overcrowding and precariousness are observed” (Razmilic, 2019: 101). In relation to the housing market, migrants must face discrimination, high prices for small places, requirements, and documentation they do not have which causes informal leasing practices, among other issues. According to Razmilic “overcrowding ranges around 20 percent among the immigrant population” (2019: 126) and it is a widespread problem among migrants. However, during this pandemic time, their overcrowded condition has become a social problem, but not because of the precarious living conditions they have in these places, but because of the fear of the neighboring population to get infected: the fear of the coronavirus once again is fear about the other, the migrant.

Another face of the pandemic effects over migrants is related to the border closure: some people are trapped on the border with Peru and Bolivia, because when they choose to return to their countries of origin, they find a sanitary barrier that closes the borders and leaves them in a limbo of airports, bus terminals, and temporary shelters. In a very worldwide view there are too many factors increasing migrants’ vulnerability to COVID-19 (Guadagno, 2020) related to increased likelihood of contracting it, not accessing appropriate care, showing severe symptoms, suffering psychosocial impacts, livelihood and income insecurity. Guadagno has also written: “… border closures have made it virtually impossible for incoming asylum seekers to apply for international protection …”(p. 11) to show the urgency that some special situations such as asylum can generate in makeshift shelters at the borders.

Specific mention has put the Food and Agriculture Organization of the United Nations around the migrant workers (FAO, 2020) because of their substantial role in agri-food systems but their vulnerability is still around any border.

The greatest threat to migrants is invisibility and discrimination by Chilean society. ¿Why? because until now, the government policies and actions established to face the social and economic effects of the pandemic, apparently do not intend to include the entire migrant population. We should not forget the significant number of migrants who are undocumented, and who – therefore – do not have the necessary documentation to apply for any kind of social benefit. This keeps them invisible in the eyes of social policy, even though they are forced to work in quarantine to survive, even though they are told that they must maintain social distance even when they must use public transportation, just to be “good inhabitants” of this country.

As in any migrants crisis around the world (Turkish, Kurdish and so on in Greece; Salvadoran, Guatemalan and so on in Mexico; among others) the non-recognized and non-visible Chilean migratory crisis is full of contradictions and demands new theoretical, humanistic, methodological and pragmatic approaches to develop situated social policies. We think that all the social situations created by the migrants moves and relationships with the receiving society must be seen as a negotiated order in the ways of symbolic interactionism legacy (Forte, 2004a, 2004b). In this challenge, social work as a discipline should play a key role.

## References

[bibr1-1473325020973363] FAO (Food and Agriculture Organization of the United Nations) (2020) Migrant workers and COVID-19 pandemic. Available at: http://www.fao.org/3/ca8559en/CA8559EN.pdf (accessed 1 July 2020).

[bibr2-1473325020973363] Forte J (2004a) Symbolic interactionism and social work: A forgotten legacy, part 1. Families in Society 85(3): 391–400.

[bibr3-1473325020973363] Forte J (2004b) Symbolic interactionism and social work: A forgotten legacy, part 2. Families in Society 85(4): 521–530.

[bibr4-1473325020973363] Gaete A Iturrieta P Peña C, et al. (2019) The 2019 social outbreak in Chile: Evidence of a delusional oasis.

[bibr5-1473325020973363] Guadagno L (2020) Migrants and the COVID-19 Pandemic: An Initial Analysis. Migration Research Series 60. Geneve: International Organization for Migration.

[bibr6-1473325020973363] Mbembe A (2003) Necropolitics. Public Culture 15(1): 11–40.

[bibr7-1473325020973363] National Foundation for Overcoming Poverty (2017) Calidad del empleo asalariado en población migrante de la región metropolitana. (Quality os salaried employement in the migrant population of the Metropolitan region) (Available at: www2.superacionpobreza.cl/wp-content/uploads/2019/06/01_MP_Metropolitana_Calidad-del-empleo-en-la-población-migrante.pdf (accessed 1 July 2020).

[bibr8-1473325020973363] National Institute of Statistics (INE) (2019) Report. Available at: www.extranjeria.gob.cl/media/2019/07/Estimación-Población-Extranjera-en-Chile.pdf (accessed 23 June 2020).

[bibr9-1473325020973363] Razmilic S (2019) Inmigración, Vivienda y Territorio (Immigration, housing and territory). In: Aninat I and Vergara R (eds) Inmigración en Chile. Una mirada multidimensional (Immigration in Chile. A Multidimensional View). pp.101–148. CEP-FCE: Santiago, Chile.

